# Spinach Plants Favor the Absorption of K^+^ over Na^+^ Regardless of Salinity, and May Benefit from Na^+^ When K^+^ is Deficient in the Soil

**DOI:** 10.3390/plants9040507

**Published:** 2020-04-15

**Authors:** Jorge F. S. Ferreira, Jaime Barros da Silva Filho, Xuan Liu, Devinder Sandhu

**Affiliations:** 1 US Salinity Laboratory (USDA-ARS), 450 W. Big Springs Rd., Riverside, CA 90001, USA; xuan.liu@ars.usda.gov (X.L.); devinder.sandhu@ars.usda.gov (D.S.); 2 Department of Botany and Plant Sciences, University of California at Riverside, 900 University Ave., Riverside, CA 90001, USA; jaimeba@ucr.edu

**Keywords:** *Spinacia oleraceae*, Na^+^:K^+^ relation, Cl^−^:NO_3_^−^ relation, salinity-driven mineral imbalance, salt-tolerant glycophyte

## Abstract

Two spinach (*Spinacea oleracea* L.) cultivars were evaluated for their response to deficient (0.25 mmol_c_ L^−1^ or 0.25 K) and sufficient (5.0 mmol_c_ L^−1^ or 5.0 K) potassium (K) levels combined with salinities of 5, 30, 60, 90, and 120 mmol_c_ L^−1^ NaCl. Plants substituted K for Na proportionally with salinity within each K dose. Plants favored K^+^ over Na^+^, regardless of salinity, accumulating significantly less Na at 5.0 K than at 0.25 K. Salinity had no effect on N, P, and K shoot accumulation, suggesting that spinach plants can maintain NPK homeostasis even at low soil K. Ca and Mg decreased with salinity, but plants showed no deficiency. There was no Na^+^ to K^+^ or Cl^−^ to NO_3_^−^ competition, and shoot biomass decrease was attributed to excessive NaCl accumulation. Overall, ‘Raccoon’ and ‘Gazelle’ biomasses were similar regardless of K dose but ‘Raccoon’ outproduced ‘Gazelle’ at 5.0 K at the two highest salinity levels, indicating that ‘Raccoon’ may outperform ‘Gazelle’ at higher NaCl concentrations. At low K, Na may be required by ‘Raccoon’, but not ‘Gazelle’. This study suggested that spinach can be cultivated with recycled waters of moderate salinity, and less potassium than recommended, leading to savings on crop input and decreasing crop environmental footprint.

## 1. Introduction

Soil quality and water salinity are major problems for the environment and for food production. Salinity occurs in arid and semi-arid zones of every continent, except Antarctica [1]. As both water and soil salinity increase, water and nutrients absorbed by plants decrease due to osmotic effects. Thus, under saline conditions, important nutrients remain dissolved in the soil matrix, where they may be lost to the environment through leaching.

Salts present in saline soils can occur typically, naturally or as residue of fertilizers, as Na^+^, Cl^−^, SO_4_^2−^, HCO_3_^−^, and salts of Ca, Mg, and K. At moderate salinity, Na accumulates in most plant tissues at similar concentrations as N and K but is not considered an essential nutrient for glycophytic plants. At moderate to high salinity, Na and Cl accumulate in concentrations that often exceed those of macronutrients [2]. However, Na is required for growth and development by some plants, such as some C4 species [3]. As early as 1960, Na was found to be necessary for efficient growth of halophytic plants, such as halogenton [4], and more recently for *Atriplex acanthocarpa* (a saltbush species) [5]. In glycophytic plants, Na is preferentially absorbed compared to K and, under salinity, Na^+^ inhibits K^+^ uptake and displaces K^+^ from its cellular binding sites [6] and can become cytotoxic. Although most plants accumulate more Na and less K under increasing salinity, some can regulate their foliar K concentrations and a high cytosolic K^+^/Na^+^ ratio is associated with a higher degree of salt tolerance [7,8]. However, the K^+^/Na^+^ ratio in shoots may not be a useful parameter to evaluate salinity tolerance or toxicity of these plants because shoot Na may increase with salinity while K remains stable.

Potassium (K) is recognized as a major macronutrient playing several important roles in plant physiological and metabolic processes; K is also associated with increased plant tolerance to biotic and abiotic stresses, including salinity [9,10]. These authors have shown that providing K in excess of the recommended crop dose reduced Na absorption and increased K accumulation in plant tissues. While Na is partly unable to replace K in plants in its vital roles as enzyme co-factor, charge balance, osmotic function, and mainly protein synthesis [11], halophytic plants seem to benefit from NaCl accumulation in their cell vacuoles as osmoticum and improve plant growth. The prevailing consensus is that glycophytic plants cannot benefit from Na to increase their growth. However, sugar beet (*Beta vulgaris*), a glycophyte with lower sensitivity to Na, belonging to the Chenopodiaceae (Amaranthaceae *sensu lato*) like spinach, was reported to replace 75% to 95% of leaf K by Na, while still increasing shoot biomass [12]. Later, growth of sugar beet was reported to be unaffected up to the substitution of 99.75% of K by Na, applied as NaCl [11]. Similar studies have not been performed for other glycophytic members of this family, such as spinach. 

Spinach (*Spinacea oleracea* L.), a glycophytic C3 species, has been reported by some as moderately-tolerant to NaCl if cultivated in the winter, but sensitive to moderately-sensitive if cultivated in the spring and summer [13,14,15,16]. Speer and Keiser (1991) reported little effect of 100 mM NaCl on spinach growth after 17 days of irrigation [17]. Although Na cannot replace K in its metabolic and physiological roles, a few authors have reported that when Na was absent from the growing medium, spinach plants grew poorly [18,19]. Interestingly, recent studies, using Ca(NO_3_)_2_ as source of N instead of NaNO_3_, reported higher spinach biomass when NaCl was added to the irrigation water [15,20]. In contrast, another recent study found growth of ‘Crocodile’ spinach to be greatly reduced by irrigation-water salinity of EC_w_ = 6.5 dS m^−1^ [21]. Thus, the literature provides conflicting results on the salinity tolerance of spinach and very little on the role of Na in spinach growth.

Although Na may improve the growth of certain glycophytic plants such as red beet, it has not been established as an essential mineral for spinach. Additionally, moderate and high salinities of soil or irrigation water are considered to lead to mineral nutrient imbalance caused by excess NaCl, carbonates, sulfates, and salts of Ca, Mg and K [1,2]. These excess salts, mainly Na and Cl, are known to cause antagonism between cations (e.g., Na^+^ vs. K^+^) and anions (e.g., Cl^−^ vs. NO_3_^−^), even when nutrients are provided at recommended rates, leading to reduced plant growth and yield due to mineral imbalance [2,21]. Because abiotic stresses do not happen in isolation [22] and soil K has low availability to plants [23], salinity may aggravate K deficiency and the general plant mineral status [24]. According to the latter authors, tomato plants could maintain leaf growth for a week under K^+^ starvation or salt stress alone, but not in combination. These authors also stressed that research on combined stresses will make a substantial contribution to maintaining global food production. As we could not find reports on spinach growth under salinity and potassium deficiency stresses combined, we deemed it important to evaluate spinach yield and the relationship of Na vs. K and Cl vs. N through their tissue accumulation. Thus, our objectives were to evaluate: (1) the relationship between NaCl-induced salinity vs. macronutrient tissue accumulation in two commercial spinach cultivars; (2) spinach biomass accumulation under the combination of salinity and K deficiency; (3) possible competition between Na vs. K and Cl vs. N imposed by increasing tissue accumulation of Na and Cl.

We hypothesized that spinach growth was going to be significantly reduced in response to high salinity and potassium deficiency, mainly when combined through the irrigation water. We also hypothesized that Na and Cl tissue accumulation would be antagonistic to K and N tissue accumulation, respectively. 

## 2. Materials and Methods

### 2.1. Plant Cultivation and Makeup of Saline Waters

The experiments were conducted from March 16 (seeding) to May 15 (harvest) 2018 in a greenhouse with two spinach cultivars, Raccoon and Gazelle (Johny Seeds, Fairfield, ME, USA), in Riverside, CA (Lat. 33.9°58′24″ N, Long. 117°19′12″ E, Alt. 311 m). During that period, greenhouse temperature was maintained at 25–33 °C (days)/14–23 °C (nights) under natural illumination (Figure S1). Minimum relative humidity (RH) ranged from 10% to 32% while maximum RH ranged from 39% to 47%, and intensity of natural light ranged from 650 to 1225 µmol m^−2^ s^−1^ (Figure S1).

Seven seeds were sown directly into pots of 22.2-cm in diameter and 1.5-gallon (7.2-L) capacity filled with one-part non-washed, non-sterile sand and one-part local soil (sandy loam). After sowing (18 days), germinated seedlings were thinned to three size-homogeneous plants per pot. From seeding to the 4-true-leaf stage, plants were watered daily with deionized water. Seven days after thinning, at the growth stage of 4 true leaves, plants were watered with 150.0 mL per pot of ½ Hoagland’s solution daily. This basic-nutrient solution contained (in mmol_c_ L^−1^): Ca (NO_3_)_2_ (2.25), NaNO_3_ (3.5), NaH_2_PO_4_ (0.5), MgSO_4_ (1.0), Fe (0.05) as sodium ferric diethylenetriamine pentaacetate (NaFe-EDTA), H_3_BO_3_ (0.023), MnSO_4_ (0.005), ZnSO_4_ (0.0004), CuSO_4_ (0.0002), and H_3_MoO_4_ (0.0001). However, plants assigned to the sufficient-K treatment received (in mmol_c_ L^−^^1^): KNO_3_ (3.5), instead of NaNO_3_ and KCl (1.25). Plants assigned to the low-potassium (K) treatment received no supplemental K. Those plants had only the K available in the soil. Chemical analysis of the sand:soil mixture determined that K^+^ was present at 0.25 mmol_c_ L^−1^ (250 µM), like the 100 µM K^+^ previously established as the deficient-K soil dose in a gene expression analysis in Arabidopsis [25].

Salinity treatments comprised of five NaCl (referred by their nominal concentrations of 5, 30, 60, 90, and 120 mmol_c_ L^−1^ of Na^+^ or Cl^-^) and two K^+^ (0.25 and 5.0 mmol_c_ L^−1^) concentrations were constructed using Extract Chem v. 2.0 [26] with electrical conductivity of irrigation water (EC_iw_) ranging from 1.3–1.6 dS m^−1^ (control) to 13 dS m^−1^ in the highest salinity treatment (Table 1). All solutions were based on a modified ½-strength Hoagland’s as basic nutrition solution, except those treatments assigned to low-K, which received solutions without K and were limited to what was available in the growing medium, as specified above. The salt composition of irrigation waters shown in Table 1 was balanced for cations and anions to assure the salts remained in solution. However, the control T2–0 (5.0 mmol_c_ L^−1^ K) had a Na concentration lower than T1–0 (0.25 mmol_c_ L^−1^ K) to balance a higher K concentration of 5.0 mmolc L^−1^ but contained macro and micronutrients at the same concentrations as the other solutions. As specified above, Na was included (at 3.0–6.5 mmol_c_ L^−1^) to ½-strength Hoagland’s control solutions to balance cations. However, our previous experiment with ‘Raccoon’ spinach showed that low concentrations of NaCl, as used in our control solutions, had no impact on spinach biomass [27].

### 2.2. Experimental Setup and Statistical Analysis

Pots in each cultivar were arranged in a complete randomized design in a split-plot arrangement. The experiment comprised of 2 spinach cultivars, with 2 K doses (main plot) combined with 5 NaCl concentrations (sub-plot), and 4 replicates per treatment (each with 3 plants pot^−1^) with a total of 40 pots per cultivar. Data for shoot biomass and the accumulation of minerals (including Na and Cl), was analyzed with ANOVA and regression for shoot dry biomass. Means were compared by the Fisher’s LSD test (*p* < 0.05). 

### 2.3. Application of Salinity Treatments

Irrigation with salinity treatments commenced when plants had six to eight true leaves (two weeks after thinning) and continued for 28 days until harvest. On 16 April 2018, pots were saturated with the modified ½-strength Hoagland’s solution (low-salinity control). From 18 to 24 April, salinity treatments were applied by watering pots with 300 mL of pertinent saline water treatment to allow a leaching fraction (LF) of 20%. On 25 April, LF was adjusted to approximately 30% by adding 500 mL of pertinent saline irrigation per pot. From 27 April to 6 May, irrigation volume increased to 400 mL per pot to maintain leaching fraction, and from 7 to 15 May, irrigation volume was maintained at 500 mL per pot to maintain a 30% LF. The irrigation volumes were increased to maintain a constant LF while adjusting to plant growth.

### 2.4. Plant and Soil Collection for Analysis

Plants were harvested on May 16, 2018, washed with tap water, then deionized water, to remove any mineral impurities from irrigation left on leaves. Leaves were blotted dry with paper towels and immediately dipped into liquid nitrogen for about 1.5 min. Frozen samples were kept at −20 °C until freeze-dried at −52 to −55 °C in a Freeze Dry System (FreeZone 6, Labconco, Kansas City, MO, USA) for 72 h. Freeze-dried samples were separated into shoots and roots, weighed to record dry mass, then ground in a Wiley mill to pass a 20-mesh (0.84 mm) screen. Tissue mineral concentration was based on shoot dry weight (DW). Chloride was determined from nitric-acetic acid extracts by amperometric titration. The concentrations of Na, the macronutrients P, K, Ca, Mg, and total-S, and of the micronutrients Fe, Cu, Mn, Zn, Se, and Mo were determined from nitric acid digestions (Milestone, Ethos EZ Microwave Digestion, Shelton, CT, USA) of the dried, ground, plant material by Inductively Coupled Plasma Optical Emission Spectrometry (ICP-OES, 3300DV, Perkin-Elmer Corp., Waltham, MA, USA). Nitrogen was determined by combustion in a Rapid N Exceed^®^ analyzer (rNex, Elementar Americas Inc., Ronkonkoma, NY, USA). After plants were harvested, three soil samples per pot were taken from each of the four replicates at 10 cm from soil surface using a small hand shovel and combined as one soil sample per replicate. Each soil sample weighed about 340 g and its saturation paste and extracts were analyzed by ICP-OES as described for leaves. The electrical conductivity of the soil-paste extract (EC_e_) was measured by an EC meter (Amber Science Inc. Model 1056, Eugene, OR, USA) and the pH was measured by a pH meter (Orion VersaStar, ThermoFisher, Waltham, MA, USA).

## 3. Results

### 3.1. Salinity Effects on Soil Na, Cl, K, and NO_3_^−^

Plants were grown in 1:1 (sand:loamy sand soil) containing 0.25 mmol_c_ L^−1^ of K with no extra K provided for the low-K treatment (0.25 K). In the treatment with sufficient K (5.0 mmol_c_ L^−1^), K was provided in the irrigation water. At the end of the experiment, soil-paste analysis revealed that Na and Cl increased drastically from control salinity at 0.25 K (T1–0) and 5.0 K (T2–0) (EC_iw_ = 1.3 dS m^−1^) to the highest salinity at 0.25 K (T1–4) and 5.0 K (T2–4) (EC_iw_ = 13 dS m^−1^). Increase in Na^+^ and Cl^−^ in the soil paste, at 0.25 K, from T1–0 to T1–4 (‘Raccoon’) was about 23- and 36-fold, respectively, and from T1–0 to T1–4 (‘Gazelle’) it was about 19-fold (Na^+^) and 38-fold (Cl^−^), respectively. Increase in Na^+^ and Cl^−^ in the soil paste, at 5.0 K, from T2–0 to T2–4 (‘Raccoon’) was about 44-fold (Na^+^) and 27-fold (Cl^−^), respectively, and from T2–0 to T2–4 (‘Gazelle’) it was about 47-fold (Na^+^) and 61-fold (Cl^−^), respectively (Table 2). Soil NO_3_^−^ (for ‘Raccoon’, but not ‘Gazelle’) and K^+^ also accumulated in the soil paste by 3.0–3.8-fold at 0.25 K, and 1.3–1.5-fold at 5.0 K, respectively, as salinity increased. While Na^+^ and Cl^+^ in soil paste increased similarly to increased concentrations in irrigation water (Table 1) and macronutrients remained in the soil at ½ to ⅓ the concentration provided by irrigation water, NO_3_^−^ had the highest reduction in soil paste remaining from ½ to ⅕ of its concentration in irrigation water (Table 1 and Table 2). Micronutrients remained stable in soil paste samples from all treatments, but Mn had a 3.0-fold increase (at 0.25 K) and 1.4-fold increase (at 5.0 K) with salinity in ‘Raccoon’. Soil-paste electrical conductivity (EC_e_) increased from its original EC_e_ of 0.5 dS m^−1^ (see Table 1 footnote) to at least 1.0 dS m^−1^ in control salinity and to 20-fold its original value at the highest salinity, regardless of K dose (Table 1 and Table 2). Soil EC_e_ was similar to the irrigation water EC_iw_ up to 30 mmol_c_ L^−1^. Then, EC_e_ dropped in average 1.4, 2.0, and 2.6 EC units when salinity rose to 60, 90, and 120 mmol_c_ L^−1^, respectively (Table 2).

### 3.2. Shoot Na and Cl Concentrations and Their Relation to K

**Figure 1:**
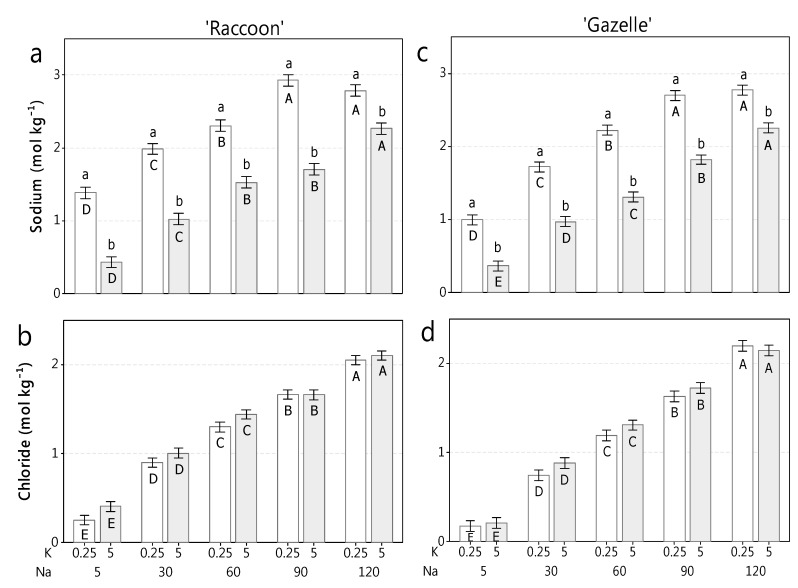
Shoot ion concentrations in spinach cultivars. Shoot sodium (**a**) and chloride (**b**) for cv. Raccoon, and sodium (**c**), and chloride (**d**), for cv. Gazelle as function of potassium (K) doses (0.25 and 5.0 mmol_c_ L^−1^) within each salinity (Na) level (from 5 to 120 mmol_c_ L^−1^ of NaCl). Mean bars with different letters in the same sodium (Na) concentration are significantly different from each other by Fisher’s LSD test (*p* ≤ 0.05). Bars represent standard errors of means (*n* = 4). K available for plants were 0.25 (from soil) and 5.0 mmol_c_ L^−1^ (provided with irrigation water). Na concentrations (in mmol_c_ L^−1^) were 5.0, 30, 60, 90, and 120. Lowercase letters show significant difference (*p* < 0.05) between K doses within the same Na level, while uppercase letters show significant differences (*p* < 0.05) among different salinity levels within the same K dose.

Shoot concentration of Na increased significantly and sharply when salinity increased, both at 0.25 K (0.25 mmol_c_ L^−1^) and at 5.0 K (5.0 mmol_c_ L^−1^) (Figure 1). At 0.25 K, ‘Raccoon’ increased shoot Na over 2-fold from 1.4 to 2.95 mol kg^−1^ (32–68 g kg^−1^) as salinity increased, while ‘Gazelle’ increased Na 2.7-fold from 1.0 to 2.7 mol kg^−1^ (23–62 g kg^−1^). At 5.0 K, ‘Raccoon’ increased shoot Na 5.2 fold from 0.43 to 2.26 mol kg^−1^ (10–52 g kg^−1^), while ‘Gazelle’ increased Na 5.8-fold from 0.4 to 2.26 mol kg^−1^ (9–52 g kg^−1^). Both cultivars had maximum Na concentrations ranging from 2.26 to 2.95 mol kg^−1^ (52–68 g kg^−1^) for ‘Raccoon’ and 2.26 to 2.7 mol kg^−1^ (52–62 g kg^−1^) for ‘Gazelle’ (Figure 1).

Shoot Cl increased 8-fold from 0.25–2.0 mol kg^−1^ (9–72 g kg^−1^) in ‘Raccoon’ and 13-fold (0.17–2.2 mol kg^−1^ or 6–78 g kg^−1^) in ‘Gazelle’ at 0.25 K, while at 5.0 K shoot Cl increased 5.3-fold from 0.4–2.1 mol kg^−1^ (14–74 g kg^−1^) in ‘Raccoon’ and 9.5-fold (0.22–2.14 mol kg^−1^ or 8–76 g kg^−1^) in ‘Gazelle’ (Figure 1b,d). Unlike Na, Cl absorption did not diminish with increased K and, at the highest salinity level, averaged 2.06 mol kg^−1^ (73 g kg^−1^) in ‘Raccoon’ and 2.17 mol kg^−1^ (77 g kg^−1^) in ‘Gazelle’. Neither cultivar showed a difference in Cl accumulation under 0.25 K or 5.0 K, regardless of potassium dose (Figure 1b,d).

**Figure 2:**
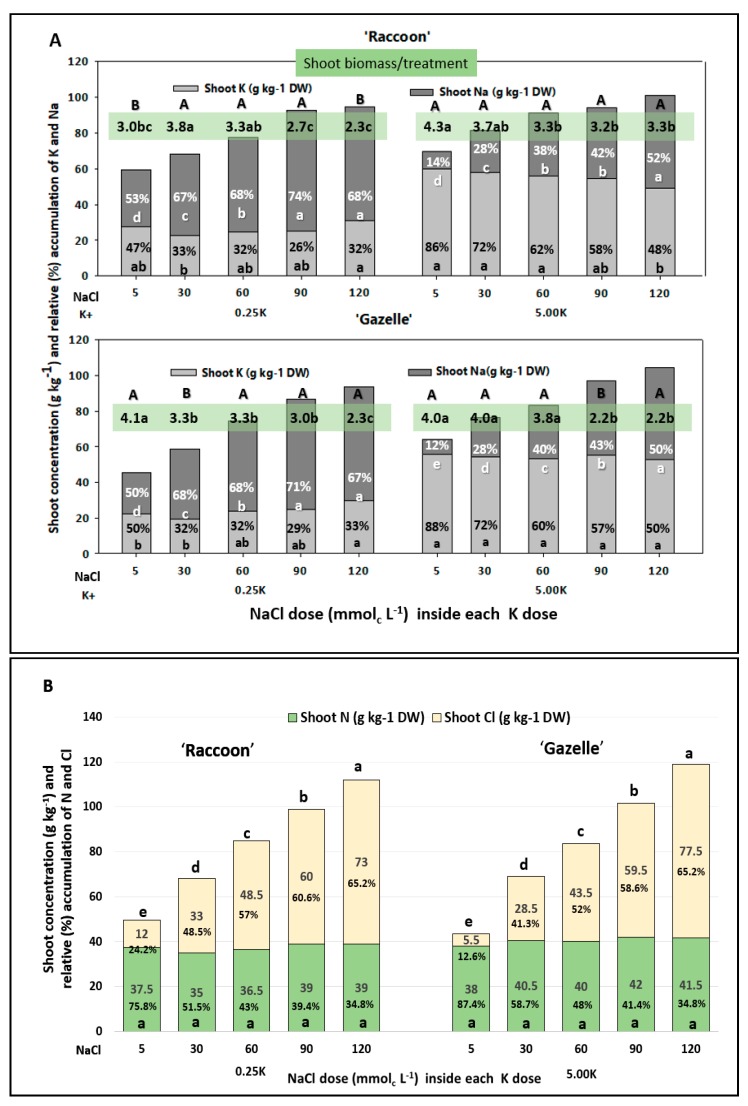
Concentrations (g kg^−1^) of (**A**) shoot potassium (K) and sodium (Na) and (**B**) shoot nitrogen (N) and chloride (Cl) of ‘Raccoon’ and ‘Gazelle’, and their relative shoot accumulations (in %) under different combinations of K and NaCl, within each K dose (0.25 K and 5.0 K). Lower-case letters show significant differences between shoot concentrations (g kg^−1^), not relative % shoot accumulation, of K, Na, N, or Cl for each NaCl dose, within each K dose. Data inside green rectangles in (**A**) show dry shoot biomass (DSB) per NaCl treatment combined with each K dose, with lower-case letters showing significance in DSB between two NaCl treatments inside the same K dose, while upper-case letters show significance in DSB between the same NaCl treatment (5–120 mmol_c_ L^−1^) in different K doses. Relative shoot concentrations (%) shows similarities in how cultivars accumulate K, Na, N, and Cl across NaCl doses and in each K dose and are not meant for statistical comparison.

In relation to K, saline treatment solutions provided 95–99.8% Na at 0.25 K and 33–95.8% Na at 5.0 K (Table 1). However, shoot tissues accumulated only 53–74% Na in relation to K at 0.25 K and 14–52% Na in relation to K at 5.0 K in ‘Raccoon’ and 50–71% Na in relation to K at 0.25 K and 12–50% Na in relation to K at 5.0 K in ‘Gazelle’ (Figure 2).

### 3.3. Mineral Leaf Composition in Response to NaCl and K Doses

**Figure 3:**
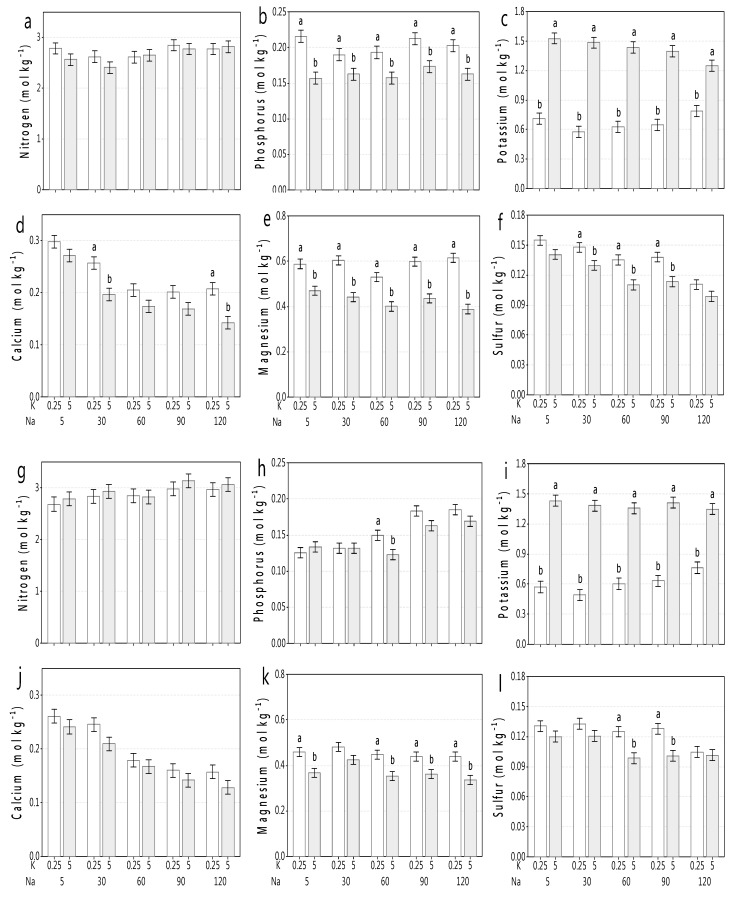
Ion concentrations of shoot macronutrients in two spinach cultivars grown under different salinity (Na) and potassium (K) treatments. ‘Raccoon’ shoot concentrations of N (**a**), P (**b**), K (**c**), Ca (**d**), Mg (**e**), and S (**f**) and ‘Gazelle’ shoot concentrations of N (**g**), P (**h**), K (**i**), Ca (**j**), Mg (**k**), and S (**l**). Means followed by different letters show significant differences between K doses in each Na dose by Fisher’s LSD test (*p* < 0.05).

Shoot macronutrients were generally not affected by salinity, except Ca and to a minor extent S. Similarly, K doses in general did not affect shoot macronutrient concentration, except for P and Mg, which were reduced at the higher dose of K (5.0 mmol_c_ L^−1^) (Figure 3). However, these lower levels of P, Ca, Mg, and S were still adequate for plant growth. Thus, we restricted our results (and discussion) to the main macronutrients NPK, mainly N and K as their source ions NO_3_^−^ and K^+^ are said to compete with Cl^−^ and Na^+^, respectively, under conditions of elevated salinity.

‘Raccoon’ maintained N, P, and K with increased salinity (Figure 3a–c). Foliar N did not change across salinity or with K doses and ranged from 2.5 to 2.85 mol kg^−1^ (35 to 40 g kg^−1^) in ‘Raccoon’ and from 2.7 to 3.0 mol kg^−1^ (38 to 42 g kg^−1^) in ‘Gazelle’ (Figure 3a,g). In neither cultivar there was any cumulative competition between Cl and N in shoots. However, shoot P concentrations were significantly lower when K dose increased and ranged from 0.19 to 0.22 mol kg^−1^ (5.9 to 6.7 g kg^−1^) at 0.25 K and from 0.16 to 0.17 mol kg^−1^ (4.9 to 5.1 g kg^−1^) at 5.0 K, while ‘Gazelle’ ranged from 0.13 to 0.18 mol kg^−1^ (3.9 to 5.6 g kg^−1^) at 0.25 K and from 0.13 to 0.16 mol kg^−1^ (4.0 to 5.0 g kg^−1^) at 5.0 K (Figure 3b,h). Although P significantly decreased when K increased in ‘Raccoon’, but not in ‘Gazelle’ (Figure 3b,h), P significantly increased at the two highest salinity levels in ‘Gazelle’ for both 0.25 K and 5.0 K (Figure 3h). Shoot K concentration increased significantly and substantially (approximately two-fold) when K increased from 0.25 to 5.0 mmol_c_ L^−1^. Foliar K ranged from 0.72 to 0.77 mol kg^−1^ (28–30 g kg^−1^) for ‘Raccoon’ at 0.25 K and from 1.28 to 1.54 mol kg^−1^ (50–60 g kg^−1^) at 5.0 K with a similar range for ‘Gazelle’ plants at each K dose (Figure 3c,i).

Shoot concentration of Ca decreased significantly with salinity for both cultivars, regardless of K dose. Ca decreased between control (5 mmol_c_ L^−1^) and the highest salinity (120 mmol_c_ L^−1^) treatment by 33.3% (at 0.25 K) and by 45.5% (at 5.0 K) in ‘Raccoon’ (Figure 3d) and by 45.5% (0.25 K) and 49% (5.0 K) in ‘Gazelle’ (Figure 3j). While Mg decreased significantly for both cultivars when K increased from 0.25 K to 5.0 K, regardless of salinity, Mg remained fairly constant across salinity for both cultivars, averaging 0.55 mol kg^−1^ (13.5 g kg^−1^) for ‘Raccoon’ and 0.46 mol kg^−1^ (11.25 g kg^−1^) for ‘Gazelle’ at 0.25 K, but leaf concentrations reduced significantly with K = 5.0 mmol_c_ L^−1^ in approximately 0.08–0.24 mol kg^−1^ (2.0–6.0 g kg^−1^) for both ‘Raccoon’ and ‘Gazelle’ (Figure 3e,k).

Sulfur decreased with both salinity and K dose, but not always consistently (Figure 3f,l). 

A decrease in S (31%) was observed with increased salinity in ‘Raccoon’, under both K doses, while the decrease was less accentuated (ranging from 16–21%) for ‘Gazelle’. However, the decrease in S was most heightened at NaCl concentrations of 60–120 mmolc L^−1^ in both cultivars (Figure 3f,i).

### 3.4. Interaction among Potassium, Salinity, and Micronutrients

Micronutrient leaf concentrations varied widely with salinity, K doses, and cultivars. However, significant decreases in micronutrients were mostly observed in shoots of ‘Raccoon’ (Table S1) rather than ‘Gazelle’ (Table S2). Cu decreased (although not always significantly) when K dose increased, mainly at the three highest salinity levels but had a less marked decrease with salinity. Mn and Fe remained constant with a salinity increase for both cultivars, but Mn decreased significantly at every salinity level for ‘Raccoon’ when K dose increase, but only at 60 and 120 mmol_c_ L^−1^ for ‘Gazelle’ (Table S1 and S2). Fe decreased significantly from 0.25 K to 5.0 K from 60 to 120 mmol_c_ L^−1^ NaCl for ‘Raccoon’, but was only at 60 mmol_c_ L^−1^ for ‘Gazelle’ (Tables S1 and S2). Leaf Mo remained constant across salinity and K doses in both cultivars, suggesting that neither Na, Cl, or K had antagonistic effects on Mo shoot accumulation. Leaf Se remained stable across salinity in both cultivars but decreased significantly from 0.25 K to 5.0 K for ‘Raccoon’, but not for ‘Gazelle’. Zn remained stable with salinity and K dose and ranged from 20 to 62 mg kg^−1^ for ‘Gazelle’ but decreased significantly at the two highest salinity treatments in ‘Raccoon’ at 5.0 K (Table S1). 

### 3.5. Effect of NaCl and K Doses on Spinach Biomass 

**Figure 4:**
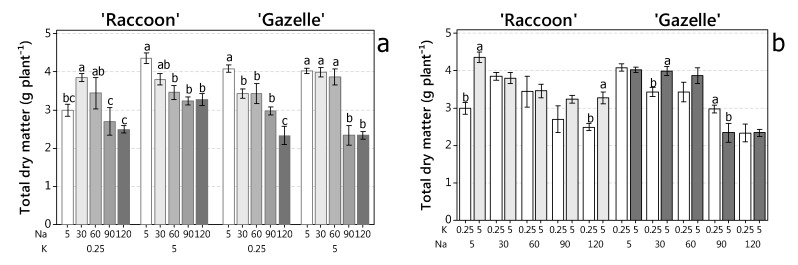
Total dry biomass (roots + shoots) of ‘Raccoon’ and ‘Gazelle’ spinach plants cultivated in a greenhouse and irrigated with saline waters. (**a**) Total biomass response to salinity within each K dose. (**b**) Total biomass response to K doses within each salinity level. Bars followed by the same letter are not different within each K (**a**) or NaCl (**b**) doses, according to Fisher’s LSD test (*p* < 0.05).

In general, and inside each K dose, salinity led to a significant decrease in total dry matter, while K dose showed an effect on plant growth at 5, 90, and 120 mmol_c_ L^−1^ NaCl for ‘Raccoon’ and at 30 and 60 mmol_c_ L^−1^ NaCl for ‘Gazelle’ (Figure 2 and Figure 4). Results for shoot plus root biomass are presented as total dry matter because root dry biomass was a fraction of shoot dry weight and remained constant for both cultivars, regardless of K dose and salinity (data not shown). At 0.25 K, a significant decrease in total dry matter was observed for ‘Raccoon’ at 90 and 120 mmol_c_ L^−1^ NaCl while for ‘Gazelle’, the decrease was significant from control to 30–90 mmol_c_ L^−1^ NaCl with another significant decrease at 120 mmol_c_ L^−1^ NaCl. At 5.0 K, ‘Raccoon’ had decreased shoot biomass with salinity, but the decrease was not significant from 30 to 120 mmol_c_ L^−1^ NaCl (Figure 4b). Potassium dose only increased biomass of ‘Raccoon’ significantly at the lowest and the highest salinity (Figure 4b). Total biomass of ‘Gazelle’ decreased significantly with salinity, at 0.25 K, but was maintained up to 60 mmol_c_ L^−1^ at 5.0 K (Figure 4a). Plants maintained biomass up to 30 mmol_c_ L^−1^ (‘Raccoon’) or up to 60 mmol_c_ L^−1^ (‘Gazelle’) and only ‘Raccoon’ seemed to benefit from Na to accumulate biomass when K was present at low levels (0.25 K) (Figure 4a). 

## 4. Discussion

### 4.1. Salinity Effects on Soil Na, Cl, K, and NO_3_^−^

Concentrations of Na and Cl in the growth medium were like that of irrigation water from 5 to 30 mmol_c_ L^−1^, but were 10–25 mmol_c_ L^−1^ lower (Table 2) than irrigation water (Table 1) when potassium was at 0.25 K, and only 5–14 mmol_c_ L^−1^ lower when potassium was at 5.0 K. This confirms that plants were exposed to higher Na and Cl as salinity increased. On the other hand, K and NO_3_^−^ concentrations in the soil were 2- to 3-fold higher at higher irrigation water salinities (90 and 120 mmol_c_ L^−1^ at both 0.25 K and 5.0 K), indicating that more NO_3_^−^ and K^+^ were left unabsorbed in the soil as salinity increased (Table 2). Similar observations were made for corn grown under salinity, and with unabsorbed NO_3_^−^ and K^+^ being lost with the leachate [28]. At the end of 28 days of saline irrigation, soil EC_e_ reached high values of 10.4 and 11.2 dS m^−1^ (Table 2), indicating that spinach can tolerate soil salinities at least 5-fold higher than the 2.0 dS m^−1^ reported as the species threshold salinity [14] without reducing significantly its shoot biomass or showing any symptoms of salinity toxicity or mineral deficiency. 

### 4.2. Shoot Na and Cl Concentrations and Their Relation to N and K

Plants of both cultivars acquired significantly less Na under 5.0 K compared to 0.25 K at all Na concentrations (Figure 1a,c). At control Na level, plants absorbed 2.5- (‘Gazelle’) to 3.0-fold (‘Raccoon’) less Na as K increased from 0.25 K to 5.0 K (Figure 1a,c). Even at the highest salinity levels increased K was associated with a significant drop in shoot-Na accumulation from 1.2- to 3.0-fold (Figure 1a,c). Our data clearly show that, even under moderate to high salinity, spinach plants prefer K over Na, when K is sufficient. Interestingly, Na accumulation in tissues of both spinach cultivars seemed to reach a plateau around 65 g kg^−1^ DW when salinity reached 90 mmol_c_ L^−1^.

Like Na, shoot Cl concentration increased steadily and significantly with increased salinity, regardless K dose (Figure 1b,d) but, unlike Na, did not reach a plateau at 90 mmol_c_ L^−1^ and increased significantly from 90 to 120 mmol_c_ L^−1^. The anions Cl^−^ and NO_3_^−^ and cations Na^+^ and K^+^ are said to compete when salinity increases significantly in the root environment causing a mineral imbalance (mainly of N and K) in plant organs. However, spinach plants of both cultivars maintained both N and K tissue concentrations throughout salinity increase (Figure 3a,c,g,i).

**Figure 5:**
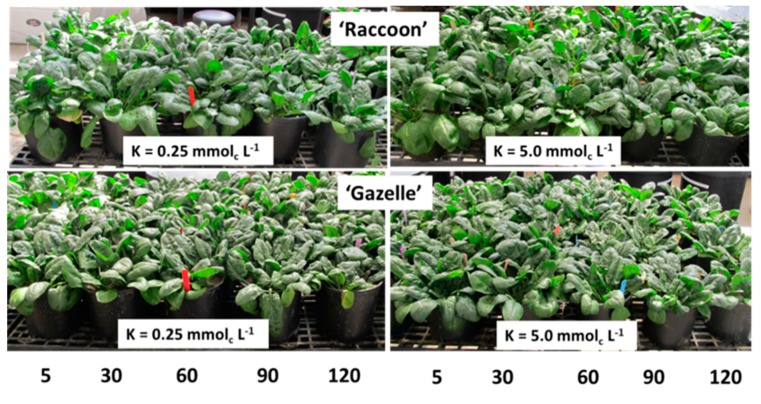
Visual aspect of ‘Raccoon’ and ‘Gazelle’ spinach cultivated for 58 days, 28 of which involved irrigation with saline waters ranging from 5 to 120 mmol_c_ L^−1^ of NaCl combined with low (0.25 mmol_c_ L^−1^) or sufficient (5.0 mmol_c_ L^−1^) potassium (K).

For both spinach cultivars, plants accumulated more shoot Na at 0.25 K compared to 5.0 K on a shoot dry weight (g kg^−1^) basis (Figure 2). Plants of both cultivars absorbed similar proportions of Na in relation to K both at 0.25 K and at 5.0 K. The fact that plants of both cultivars absorbed significantly less Na when K was sufficient, suggests that Na may have been used to maintain cell turgor and ionic homeostasis to sustain plant growth and development when K was low. However, there were no visual signs of salinity (Na or Cl) toxicity in any of the plants (Figure 5). Thus, when K was provided at 5.0 mmol_c_ L^−1^, enough for spinach growth [27], plants significantly reduced Na absorption at every salinity level (Figure 1 and Figure 2). Previously, when ‘Raccoon’ spinach received K at 3, 5, and 7 mmol_c_ L^−1^ and NaCl concentrations ranged from 2 to 80 mmol_c_ L^−1^, shoot Na increased from 4 to 28 g kg^−1^ and Cl increased from 10 to 44.6 g kg^−1^ [27], also with no decrease in N or K. 

Our results strongly suggest that spinach plants possess a mechanism that regulates Na intake-and that K absorption is favored over Na, even at high salinity, but there appears to be no mechanism to regulate Cl^−^ uptake.

Many plants show K deficiency when their shoots contain from 5 to 20 g kg^−1^ [12]. Interestingly, both ‘Raccoon’ and ‘Gazelle’ spinach plants accumulated over 20 g kg^−1^ of K even when K^+^ was provided at 0.25 mmol_c_ L^−1^ and had no visual symptoms of K deficiency, indicating that even when K^+^ is available at low concentrations spinach plants maintain K tissue levels for biomass accumulation (Figure 2 and Figure 3). Absence of K deficiency symptoms was also observed in sugar beet (a Chenopodiaceae like spinach) when most of the K was substituted by Na in plants [29]. Under low K conditions, K^+^ could be replaced by Na^+^ in its role of vacuolar charge balancing and can help maintain the cell osmoticum when K^+^ is low [30]. However, this has not been tested before with low K and high salinity combined.

### 4.3. Mineral Shoot Composition in Response to NaCl and K Doses

Spinach plants could balance most mineral nutrients to maintain growth up to 60 mM NaCl (‘Gazelle’), or slightly increase growth (‘Raccoon’) at 30 and 60 mM NaCl. Spinach shoots had from 3.5% to 4.2% N, regardless of salinity (Figure 3a,g). Thus, based on shoot accumulation of N and the fact that no other source of N (e.g., NH_4_^+^) was provided to plants, one can conclude that there was no competition between Cl^−^ and NO_3_^−^ in spinach plants, indicating that salinity does not lead to N imbalance in spinach, unlike for other glycophytic species [2]. Leaf N concentrations of 48 to 53 g kg^−1^ were reported for spinach grown with full- and half-Hoagland’s nutrition, but without salinity [31]. However, under similar salinity (EC_e_ ranging from 0.7 to 11.5 dS m^−1^) and with N ranging from 0 to 300 mg kg^−1^ of soil, shoot N ranged from 1.25% to 4.1% [32]. Their results were similar to ours showing that shoot N did not change or slightly increased as salinity increased, although they did not test K doses. A stable or increased shoot N concentration was also previously reported for Jerusalem artichoke (*Helianthus tuberosum* L.) and alfalfa (*Medicago sativa* L.) cultivated in sand and with waters of increased salinity [33,34].

Regarding P, all our plants accumulated P at 4.8 to 6.7 g kg^−1^ (‘Raccoon’) and at 3.9 to 5.7 g kg^−1^ (‘Gazelle’) (Figure 3b,h), above adequate for growth [35] and slightly under concentrations (7.7 to 10 g kg^−1^) reported for hydroponic spinach [31] and slightly above shoot-P concentrations for spinach irrigated with similar salinity treatments combined with five N doses [32]. Although salinity had no effect on shoot-P concentration, increased K reduced it significantly, albeit not enough to affect biomass accumulation. 

Shoot K accumulation in spinach shoots, as in N and P, was not affected at any salinity level and increased concentrations of Na^+^ in the irrigation waters did not inhibit K^+^ absorption by spinach plants or K accumulation in shoots (Figure 3c,i), as discussed above. Shoot K concentrations reported by others ranged from 60 to 80 g kg^−1^ for spinach cultivated in full- and half-strength Hoagland solution, respectively [31], while our 58-day-old plants provided with either 0.25 mmol_c_ L^−1^ (0.25 K) or 5.0 mmol_c_ L^−1^ (5.0 K) accumulated over 20 g kg^−1^ (at 0.25 K) and 48–60 g kg^−1^ (at 5.0 K), enough for plant growth. However, the almost two-fold increase in shoot K was not clearly associated with biomass increase in most salinity concentrations (Figure 2), suggesting that the lower K leaf concentration (0.25 mmol_c_ L^−1^) was a small (or not a) limiting factor for biomass accumulation (Figure 2 and Figure 4b) and that the decrease in biomass was rather a response to significant increases in shoot NaCl concentrations as salinity increased from control to 120 mmol_c_ L^−1^ (Figure 1). In agreement with our results, others [36] who studied NPK fertilization of baby spinach with 0, 63, 85, 127, and 148 kg of K ha^−1^, reported that leaf K ranged from 17.4 to 23.0 g kg^−1^ and also had no effect on spinach biomass accumulation. 

Plants have inward-rectifying K^+^ channels in several cells, including root cells and some have reported that K^+^ may be preferentially acquired and transported even against a strong Na^+^ concentration gradient, as reported for bean plants [37]. A study with guard cell inward-rectifying channels demonstrated that plant cells can increase tissue K at concentrations as low as 0.3 mmol_c_ L^−1^ of K [38], which is similar to the low K dose (0.25 mmolc L^−1^) used in this study. This could partially explain how spinach plants could grow under both saline conditions and low K or why plants did not always significantly increase shoot biomass when K was supplied at a concentration 20-fold higher (5.0 K). 

The interaction among nutrients is complex and highly dependent on several factors, including cultivar [2]. Our data confirm that irrigation water of 13 dS m^−1^ is not a concern for spinach mineral nutrition and growth. Spinach plants did not respond to K dose in most treatment combinations and maintained their concentrations of N, P, K, Mg, and S under salinity, suggesting that genetic mechanisms involved in mineral homeostasis under salinity are involved and must be further studied to better understand how glycophytic species like spinach can sustain growth under both salinity and K deprivation.

Foliar Mg concentrations decreased with increased K dose, but were more than adequate for spinach growth at 0.25 K, according to others [31,35] at 5.0 K. Fertilized spinach was reported to maintain high ratios of sum of cations over sum of anions, under 100 mM NaCl (EC_w_ = 10 dS m^−1^), in the vacuole (3.2), cytoplasm (2.8), and apoplast (1.1) compared with control salinity (4.5, 2.3, and 1.2, respectively) [17], explaining why our spinach plants maintained good levels of K and Mg, under 120 mM NaCl. Although our foliar concentrations of Ca were similar as reported for spinach cultivated with full- and half-strength Hoagland’s nutrient solution [31]), plants still had Na-induced Ca decrease of 32–54% (Figure 3d,j), as reported for plants in general [2,39].

Although recommendations for an ideal S-leaf concentration for spinach were not found, our S values (2.0 mmol_c_ L^−1^ of S) were adequate based on previous experiments with spinach [15,27]. 

### 4.4. Interaction among Potassium, Salinity and Macronutrients 

Interactions among plant mineral nutrients are complex and increasing salinity complicates these relationships. Increasing concentrations of Na and Cl in soil or irrigation water leads to shoot and root mineral deficiencies in glycophytic plants, mainly of K, Ca, Mg, and N [2]. However, our results show that spinach maintained their general shoot macronutrient levels when NaCl increased from 5 to 120 mmol_c_ L^−1^ (EC_w_ = 1.3 to 13 dS m^−1^, EC_e_ = 1.0 to 11 dS m^−1^) (Figure 3 and Table 2). It has been reported that K^+^ soil deprivation stimulates its uptake by plants and this stimulation is usually associated with increased expression of high-affinity K transporters [40]. This increased shoot K at 0.25 K supports the hypothesis of some authors [41] that there is a close link between K^+^ homeostasis and salinity tolerance, and suggests the involvement of regulatory genetic components not yet fully explored. These authors also mentioned that as passive absorption of Cl^−^ is favored all the time and that CCC (Cation Chloride Transporter)-mediated K^+^ loading may play an important role in regulating K^+^ homeostasis in the whole plant. Furthermore, recent research on Arabidopsis [25] unveiled a new K^+^ transporter (KUP7) that is expressed in root epidermal cells, predominantly localized in the plasma membrane, which mediates K uptake and translocation to the shoots, specially under limited K^+^ conditions in the soil. The levels of K^+^ deficiency used in their experiment was 100 µM, while in ours it was 250 µM. Thus, it sounds plausible that our spinach plants may have used these genetic and physiological mechanisms to maintain shoot K at sufficient levels (20–30 g kg^−1^) for growth.

Although N is reported to have a positive interaction with P [42], our data did not show a clear interaction between N and P under neither NaCl nor K doses. Instead, as N remained constant at different salinity levels, P decreased when K increased from 0.25 K to 5.0 K in ‘Raccoon’ (Figure 3a,b), while K dose had no effect on P tissue accumulation at low salinity levels for ‘Gazelle’, but decreased P accumulation slightly when water NaCl concentration increased from 60 to 120 mmol_c_ L^−1^ (Figure 3h), while reduced shoot Ca was probably associated with Na-induced Ca antagonism, not K dose (Figure 3d,j). On the other hand, a significant decrease in shoot Mg was associated with K increase at every salinity level, strongly suggesting a higher antagonism of K with Mg compared to Na with Mg. The literature has reported on the antagonism of K with Mg and with Ca [43,44], but in the current study with spinach, the antagonism of K against Mg was almost always significant, while against Ca there was only a tendency. In the case of S, leaf concentrations decreased significantly with increased K dose and from control salinity to the highest salinity treatment for both cultivars being more accentuated when NaCl doses of 60–120 mmol_c_ L^−1^ were combined with 5.0 K (Figure 3f,l).

### 4.5. Interaction among Potassium, Salinity, and Micronutrients

Although both ‘Raccoon’ and ‘Gazelle’ plants accumulated similar proportions of Na and K when salinity increased, within each K dose, their accumulation of (and changes in) shoot micronutrients were different (Tables S2 and S3), indicating that although their mechanism for balancing Na and K, or Cl and N, was similar, their micro-ionomic response was not. Increased K triggered different responses in micronutrient accumulation in each cultivar but it is interesting to note that, Mn decreased significantly in ‘Raccoon’ with increased K dose but increased with salinity at 0.25 K and Zn only accumulated in ‘Raccoon’ plants under deficient K at the two highest salinity treatments, and Fe only under the three highest salinities (Table S1). For ‘Gazelle’, plants increased Fe significantly between control and higher salinities, regardless K, suggesting salinity was the dominant stress to drive accumulation of Fe (Table S2) These findings suggest that both ‘Raccoon’ and ‘Gazelle’ may need a higher concentration of certain micronutrients to cope with oxidative stress imposed by low K or high salinity. While Fe may have been required in excess to maintain photosynthesis, when K was low, other micronutrients may be implicated in the regulation of reactive oxygen species (ROS) and protection of cell membranes. Examples of such micronutrients include Se, involved in the production or regeneration of antioxidant enzymes such as glutathione and catalase [45]; Mn, a cofactor for the antioxidant enzyme superoxide dismutase [46]; and Zn, a scavenger of ROS and protector of cell membranes under salt stress [47]. 

### 4.6. Effect of NaCl and K Doses on Spinach Biomass

Our data partially confirm that under a limited supply of K^+^, Na^+^ can replace K^+^ in the vacuole (true for ‘Raccoon’, but not ‘Gazelle’) as an alternative inorganic osmoticum [48] but disagree with the statement of the same authors that Mg and Ca can also accumulate under low K. In general, there was no positive biomass response to K dose, except at 5 and 120 mmolc/L for ‘Raccoon’ and at 30 mmolc/L for ‘Gazelle’ (Figure 4b). Although under non-saline conditions, others reported that spinach did not respond to K doses under non-saline conditions [18,36]. Lehr (1949) showed that K had little effect on shoot dry weight (13 to 14.0 g of shoots pot^−1^ for 1 to 8% K, respectively [18]). Lehr’s data indicated that spinach biomass did not respond to K when N was provided with a Na-based fertilizer (NaNO_3_). However, shoot DW was only 10.5 g pot^−1^ when plants received 8% K but were fertilized with Ca(NO_3_)_2_ indicating that spinach responded to Na more than to K. His spinach plants also grew better with Na than with Ca at similar concentrations of NO_3_. Others who evaluated the effect of salinity on spinach growth, inadvertently [15] or purposely [18] omitted Na from control irrigation solutions and used Ca(NO_3_)_2_ and KNO_3_ as source of K, Ca, and N either to avoid the use of Na [15] or to compare NaNO_3_ with Ca(NO_3_)_2_ [18]. In both cases it is evident that Ca(NO_3_)_2_ resulted in significantly less plant growth than NaNO_3_. Others compared NaCl with KCl in red beet to see how much K could be replace by Na [12] and also reported that plants grew poorly when NaCl was replaced totally by KCl. More recently, ‘Raccoon’ spinach (a Chenopodiaceae like red beet) cultivated with 3–7 mmol_c_ L^−1^ of K showed no K effect and no reduction in biomass when NaCl was provided to all treatments up to 80 mmol_c_ L^−1^ [27]. 

Besides sugar beet and spinach, the benefits of Na (or synergism between Na and K) for biomass accumulation were reported for olive trees [49], cacao [50], and eucalyptus grown in K-deficient soil [51]. Our results showed that the benefit of Na for biomass accumulation was only seen in ‘Raccoon’ from control salinity to 30 mmol_c_ L^−1^ at 0.25 K with Na doses and ‘Raccoon’ biomass producing a R^2^ = 0.92 and a calculated maximum shoot dry biomass of 3.45 g/plant for a dose of 29.18 mmolc L^−1^ of Na ($\hat{Y}=2.2474+0.4462*\sqrt{X}-0.0413*X,\;where\;*:\;significant\;at\;0.05\;by\;t-\mathrm{Test})$. However, at the same dose of K (0.25 mmol L^−1^), a linear effect ($\hat{Y}=3.7763-0.0127**X,\;\;r^{2}=0.93,\;where**:\;significant\;at\;0.01\;by\;t-\mathrm{Test})\;\mathrm{was}\;\mathrm{observed}\;\mathrm{for}\;‘\mathrm{Gazelle}’.$ In general, both cultivars sustained good biomass growth up to 60 mmol_c_ L^−1^, but reduced their biomass significantly at 90 and/or 120 mmol_c_ L^−1^ at 0.25 K. At 5.0 K, ‘Raccoon’ had no significant biomass decrease from 30 to 120 mmol_c_ L^−1^ NaCl, but biomass was reduced at all saline treatments after control, while ‘Gazelle’ sustained its biomass up to 60 mmol_c_ L^−1^ if supplied enough K (Figure 4a). Biomass accumulation differences between cultivars could stem from their differential tolerance to both shoot accumulation of Na (up to 52% at 5.0 K and 74% at 0.25 K) and/or Cl (up to 77.5%, regardless of K dose) (Figure 2). Glycophytic plants are reported to accumulate 1–20 g kg^−1^ of Cl [52], with tobacco accumulating 50 g kg^−1^ under 5 mM Cl^−^ [53]. Accumulation of Cl may lead to toxicity, physiological disfunction, and reduced growth and yield [52]. In a previous study, ‘Raccoon’ spinach accumulated 38 g kg^−1^ Na in roots and 44.6 g kg^−1^ in shoots under salinity of 80 mmol_c_ L^−1^ NaCl [27]. These ‘Raccoon’ plants had no significant reduction in root or shoot biomass, although they had small, but significant, reduction in photosynthesis, stomatal conductance, and transpiration rates [27].

Our data indicate that the threshold for NaCl and salinity of spinach irrigation water is between 60 to 90 mM NaCl, with an approximate EC_iw_ of 7 to 10 dS m^−1^ and a soil salinity (EC_e_) from 5.6 to 8.9 dS m^−1^ (Table 2), after which spinach biomass may decrease, depending on the cultivar. Our salinity spinach threshold matches the one reported for ‘Virofly’ spinach, with an estimated EC_e_ = 8 dS m^−1^ [32]. This spinach threshold is approximately 4-fold higher than the one reported previously by the US Salinity Lab [14] and similar to the one reported by Ors and Suarez (2016, 2017) for ‘Raccoon’ [15,20]. Thus, it appears that ‘Raccoon’, but not ‘Gazelle’, plants may use Na at macronutrient levels for growth when K is deficient. Although ‘Gazelle’ seemed more salt-tolerant than ‘Raccoon’ when K was sufficient, it had a similar response to ‘Raccoon’ in the percentage of K substituted by Na, regardless of salinity (Figure 2). This figure also shows that plants accumulated Cl in similar amounts without affecting N tissue accumulation, regardless of K dose. Thus, spinach may use Na (and maybe also Cl) as a cheap cell osmoticum to foster or maintain growth when K is deficient in soil. Thus, the common idea that excessive Na^+^ competes with K^+^ due to decreased K^+^ uptake and increased K^+^ efflux from the cell [7], and that Cl^−^ competes with NO_3_^−^ does not seem to hold for spinach plants, at least not up to 120 mM NaCl, thus going against the concept of antagonism between Na^+^ vs. K^+^ and Cl^−^ vs. NO_3_^−^ reported for other plants.

Our plants developed into healthy-looking spinach plants within two months of cultivation (Figure 5). For half of that time the plants were irrigated with waters of moderate to high salinity with EC_iw_ ranging from 1.3 to 13 dS m^−1^ and EC_e_ ranging from 1.0 to 11 dS m^−1^. 

### 4.7. Considerations on Salinity Tolerance Mechanisms of Spinach

The fact that shoot concentrations of K and N were maintained for both cultivars as salinity increased strongly indicates that spinach plants have membrane-bound transport proteins that favor K^+^ absorption over Na^+^ even when K^+^ is present at low concentrations and similar mechanisms work for N homeostasis. Previous works [25,54] reported genetic mechanisms involving KT/HAK/KUP K^+^ transporter gene families that are activated when K^+^ is deficient in the growth medium, with mechanisms also involving genes of the CCC (chloride transporters) family and SKOR channels that are activated by salt stress and are responsible for transporting K^+^ and NO_3_^−^ from root to shoots [41]. These types of genetic mechanisms, described in *Arabidopsis*, can account for K and N homeostasis under moderate to high salinity. Another probable tolerance mechanism may have involved the sequestration of both Na^+^ and Cl^−^ in vacuoles, accounting for spinach tolerance of high concentrations of Na and Cl. Our data supports those of others who reported that both Na and K have roles in osmotic regulation and that Na can actually promote growth in plants of the sodium-loving Chenopodiaceae (Amaranthaceae *sensu lato*) family [55,56]. As Na was also present in the nutritional solution (5.0 K) at the lowest level of salinity, we cannot say for sure if Na benefits also extend to when K is present at sufficient levels and that should be explored in controlled studies without Na. However, at least two previous studies on spinach [18] and sugar beet [12], both Chenopodiaceae, indicated that these plants do not grow as well in the absence of Na.

Although shoot Cl increased significantly with salinity, there was no antagonism of Cl to shoot accumulation of N, regardless of K dose or salinity. The fact that salinity caused significant shoot reductions in Ca and S, without reducing P, K, and Zn at low K indicate that the relationship between Na and K, and Na and Ca, must be given more attention when salinity is involved. 

## 5. Conclusions

Our results clearly showed that, based on mineral shoot accumulation, the competition between Na and K was mostly driven by K as tissue accumulation of Na decreased significantly under sufficient K, but Na accumulation did not affect K tissue accumulation even under K deficiency. Moreover, significant increases in tissue Cl had no effect on N accumulation in spinach shoots. Thus, plants provided with appropriate concentrations of K for normal growth will accumulate significantly less Na than plants under deficient K even when irrigated with waters of moderate salinity, as seen in this experiment. Shoot biomass of ‘Raccoon’ was maintained or increased under the combination of K deficiency and salinity up to 60 mmol_c_ L^−1^ NaCl. Although the response of ‘Gazelle’ under the same conditions was different, its shoot biomass for combined K deficiency and up to 60 mmol_c_ L^−1^ was like that of ‘Raccoon’. Under enough K^+^, ‘Raccoon’ outperformed ‘Gazelle’ in shoot biomass when salinity increased to 90 and 120 mmolc L-1, indicating that ‘Raccoon’ is more salt tolerant than ‘Gazelle’. Although salinity significantly decreased Ca and S in both cultivars, plants still accumulated enough of those minerals to sustain growth. As plants maintained their homeostasis of N, P, K, and Mg, shoot biomass decrease could have been the result of increased accumulation of both Na and Cl in shoots of both cultivars. A Na benefit on biomass accumulation was observed only in ‘Raccoon’ when K was deficient and NaCl of irrigation water was 30–60 mmol_c_ L^−1^. Our data suggest a salinity threshold for spinach irrigation water of approximately 7–10 dS m^−1^ and for soil salinity from 5.6 to 9.0 dS m^−1^, indicating that spinach plants can be cultivated with recycled waters of low to moderate salinity and with much less K than previously thought. Our findings suggest that spinach can be produced under the combined stresses of potassium deficiency and low to moderate salinity and that neither Na or Cl tissue accumulation up to 45 g kg^−1^ (ca. 2 mol kg^−1^ Na and 1.3 mol kg^−1^ Cl) resulted in a significant decrease in the shoot biomass of both cultivars. These findings can lead to savings in K fertilizers and to the efficient use of recycled waters to maintain the successful cultivation of irrigated spinach in arid and semiarid regions. Although Cl is already recognized as an essential mineral for plant growth, Na is not considered essential for glycophytic plants. Thus, considering the benefit of Na for ‘Raccoon’ but not ‘Gazelle’, under K deficiency, further studies with spinach cultivars both excluding and including Na are needed to determine if Na is a beneficial mineral for the growth of spinach when K is deficient in soil. There is also a need to study the combination of potassium starvation and the expression of KT/HAK/KUP K^+^ transporter gene families, CCC (chloride transporters) gene family, and SKOR channels to increase our understanding of how spinach can maintain the balance of most macronutrients, such as N, P, and K, when salinity increases significantly and K is present at very low levels in the soil.

## Figures and Tables

**Table 1:** Chemical composition (considering soil mineral composition) and electrical conductivity (EC_iw_) of the saline irrigation waters *. Deionized water (EC_iw_ = 0.05 dS m^−1^) was used to make all saline waters and all the treatments received ½-strength modified Hoagland’s as basic fertigation. No K was added to T1 irrigation treatments but full-strength Ca^2+^ (4.5 mmol_c_ L^−1^) was added to compensate for high Na^+^). No HCO^3−^ was added as the soil to be irrigated had a pH = 8.0.

Treatment	* K^+^	* Na^+^	* Cl^−^	PO_4_^3−^	* Ca^2+^	* Mg^2+^	* SO_4_^2−^	* NO_3_^−^	EC_iw_	pH
	mmol_c_ L^−1^								dS m^−1^	
T1_0	0.25	6.5	1.1	1.5	6.0	2.5	4.5	10.0	1.3	5.5
T1_1	0.25	30.5	25.1	1.5	6.0	2.5	4.5	10.0	4.2	5.4
T1_2	0.25	60.5	55.1	1.5	6.0	2.5	4.5	10.0	7.2	5.3
T1_3	0.25	90.5	85.1	1.5	6.0	2.5	4.5	10.0	10.4	5.3
T1_4	0.25	120.5	115.1	1.5	6.0	2.5	4.5	10.0	13.1	5.3
T2_0	5.00	3.0	2.4	1.5	6.0	2.5	4.5	10.0	1.6	5.4
T2_1	5.00	30.5	29.9	1.5	6.0	2.5	4.5	10.0	4.5	5.4
T2_2	5.00	60.5	59.9	1.5	6.0	2.5	4.5	10.0	7.6	5.4
T2_3	5.00	90.5	89.9	1.5	6.0	2.5	4.5	10.0	10.7	5.3
T2_4	5.00	120.5	119.9	1.5	6.0	2.5	4.5	10.0	13.2	5.3

* Ion concentration includes the ion concentration determined in the loamy sand soil (in mmol_c_ L^−1^) wet paste: Na^+^, 2.5; K^+^, 0.25; Ca^2+^, 1.5; Mg^2+^, 0.5; NO_3_^−^_,_ 2.0; Cl^−^, 1.1; SO_4_^2−^, 2.5; PO_4_^3−^, 0.015. Soil pH = 8.0 and pre-experimental EC_e_ = 0.5 dS m^−1^.

**Table 2:** Ionic composition (macronutrients, mmol_c_ L^−1^), pH, and soil electrical conductivity (EC_e_) of the sand:soil mixture from pots used to grow ‘Raccoon’ and ‘Gazelle’ spinach plants 50 days after sowing and 28 days after irrigation with saline waters combining salinity (NaCl), nutrients, and potassium (K) specified in Table 1.

	‘Raccoon’					‘Gazelle’				
K Doses	NaCl Doses (mmol_c_ L^−1^) Used to Irrigate Pots									
Used	5	30	60	90	120	5	30	60	90	120
**(mmol_c_ L^−1^)**	**NO_3_^−^, mmol_c_ L^−1^**									
**0.25**	1.78aA	1.32aA	1.28aA	2.96aA	5.31aA	1.51aBC	0.98aC	1.90aBC	2.30aB	5.83aA
**5**	1.43aA	1.24aA	1.29aA	1.64aA	1.83aA	2.28aAB	1.77aB	2.78aAB	3.34aA	3.34bA
	**PO_4_^−^, mmol_c_ L^−1^**									
**0.25**	0.52aB	0.50aB	0.57aB	0.69aA	0.03bC	0.46aA	0.41aA	0.59aA	0.48aA	0.00bA
**5**	0.32bB	0.53aA	0.60aA	0.52bA	0.53aA	0.33aA	0.44aA	0.50aA	0.62aA	0.65aA
	**K^+^, mmol_c_ L^−1^**									
**0.25**	0.13bA	0.25bA	0.29bA	0.33bA	0.38bA	0.13bA	0.20bA	0.22bA	0.29bA	0.33bA
**5**	1.17aD	1.56aC	2.08aB	2.36aAB	2.41aA	1.36aBC	1.19aC	1.64aB	2.29aA	2.46aA
	**Ca^2+^, mmol_c_ L^−1^**									
**0.25**	3.44bAB	3.57aAB	3.19aB	3.86aAB	4.16aA	4.11aAB	5.40aA	3.63aB	4.82aAB	3.96aB
**5**	5.45aA	3.60aB	3.59aB	4.07aB	4.43aB	5.31aA	4.39aA	4.74aA	4.32aA	4.41aA
	**Mg^+^, mmol_c_ L^−1^**									
**0.25**	1.36bA	1.25aA	1.10aA	1.28aA	1.39aA	1.66aAB	1.90aA	1.25aB	1.53aAB	1.36aB
**5**	1.89aA	1.13aB	1.12aB	1.22aB	1.29aB	2.02aA	1.43bB	1.46aB	1.29aB	1.30aB
	**SO_4_^−^, mmol_c_ L^−1^**									
**0.25**	2.24aB	2.13aB	2.64aA	2.72aA	1.91aB	2.82aA	2.77aA	2.76aA	3.11aA	1.90aB
**5**	2.57aA	1.86aB	1.86bB	1.93bB	1.87aB	2.45aA	2.17bAB	2.07bABC	1.77bBC	1.61aC
	**Na^+^,** **mmol_c_ L^−1^**									
**0.25**	4.15aE	26.97aD	48.81aC	73.51aB	95.50aA	5.03aE	30.70aD	49.33aC	79.69aB	94.69aA
**5**	2.30aE	24.68aD	50.78aC	76.42aB	101.27aA	2.01aE	29.39aD	54.31aC	76.02aB	94.35aA
	**Cl^−^, mmol_c_ L^−1^**									
**0.25**	2.67aE	24.24aD	44.73aC	71.54aB	97.07bA	2.55aE	30.68aD	47.84aC	78.11aB	94.09aA
**5**	4.18aE	24.46aD	52.07aC	81.56aB	107.67aA	1.62aE	26.84aD	54.38aC	77.74aB	97.34aA
	**pH**									
**0.25**	8.06aA	8.11aA	8.12aA	8.10aA	8.09aA	8.12aAB	8.06aB	8.09aAB	8.22aAB	8.30aA
**5**	8.07aA	8.08aA	8.09aA	8.05aA	7.97bA	8.24aA	8.26aA	8.23aA	8.04aA	8.12aA
	**EC, dS m^−1^**									
**0.25**	0.92aE	3.46aD	5.61aC	8.04aB	10.37aA	1.06aE	4.14aD	5.86aC	8.91aB	10.24aA
**5**	1.07aE	3.46aD	6.07aC	8.85aB	11.17aA	1.08aE	3.91aD	6.53aC	8.61aB	10.41aA

Lowercase letters show significance between potassium doses within each NaCl dose and each macronutrient. Uppercase letters show significance between NaCl doses, within each potassium dose and each macronutrient. Means following by same letter are not significantly different by Fisher’s LSD test (*p* < 0.05).

## Supplementary Materials

The following are available online at https://www.mdpi.com/2223-7747/9/4/507/s1, Figure S1: Temperature and humidity (a) and light intensity (b) of the greenhouse where spinach plants were cultivated from March 16 to May 15 of 2018. Intensity of natural light ranged from 650 to 1225 µmol m^−2^ s^−1^, Table S1: Concentrations of micronutrients (in mg kg^−^^1^ or ppm) on 'Raccoon' shoots in response to increasing concentrations of NaCl combined with two doses of potassium (K), Video S1: Concentrations of micronutrients (in mg kg^−^^1^ or ppm) on 'Gazelle' shoots in response to increasing concentrations of NaCl combined with two doses of potassium (K).

## References

[1] Rengasamy P. (2010). Soil processes affecting crop production in salt-affected soils. Func. Plant Biol..

[2] Grattan S.R., Grieve C.M. (1999). Salinity–mineral nutrient relations in horticultural crops. Sci. Hort..

[3] Kronzucker H.J., Coskun D., Schulze L.M., Wong J.R., Britto D.T. (2013). Sodium as nutrient and toxicant. Plant Soil.

[4] Williams M.C. (1960). Effect of sodium and potassium salts on growth and oxalate content of halogeton. Plant Physiol..

[5] Mata-González R., Abdallah M.A.B., Trejo-Calzada R., Wan C. (2016). Growth and leaf chemistry of *Atriplex* species from Northern Mexico as affected by salt stress. Arid Land Res. Manag..

[6] Marschner P., Broadley M., Buerkert A., Cakmak I., Others S. (2012). Marschner's Mineral Nutrition of Higher Plants.

[7] Shabala S., Cuin T.A. (2008). Potassium transport and plant salt tolerance. Physiol. Plant..

[8] Maathuis F.J.M., Amtmann A. (1999). K^+^ Nutrition and Na^+^ Toxicity: The basis of cellular K^+^/Na^+^ ratios. Ann. Bot..

[9] Sardans J., Peñuelas J. (2015). Potassium: A neglected nutrient in global change. Glob. Ecol. Biogeogr..

[10] Wang M., Zheng Q., Shen Q., Guo S.-R. (2013). The critical role of potassium in plant stress response. Int. J. Mol. Sci..

[11] Faust F., Schubert S. (2016). Protein synthesis is the most sensitive process when potassium is substituted by sodium in the nutrition of sugar beet (Beta vulgaris). Plant Physiol. Biochem..

[12] Subbarao G.V., Wheeler R.M., Stutte G.W., Levine L.H. (1999). How far can sodium substitute for potassium in red beet?. J. Plant Nutr..

[13] Grieve C.M., Grattan S.R., Maas E.V., Wallender W.W., Tanji K.K. (2012). Plant salt tolerance. ASCE Manual and Reports on Engineering Practice.

[14] Maas E.V., Hoffman G.J. (1977). Crop salt tolerance- current assessment. J. Irrig. Drain. Div..

[15] Ors S., Suarez D.L. (2016). Salt tolerance of spinach as related to seasonal climate. Hort. Sci. (Prague).

[16] Shannon M.C., Grieve C.M. (1999). Tolerance of vegetable crops to salinity. Sci. Hort..

[17] Speer M., Kaiser W.M. (1991). Ion relations of symplastic and apoplastic space in leaves from *Spinacia oleracea* L. and *Pisum sativum* L. under salinity. Plant Physiol..

[18] Lehr J.J. (1949). Exploratory pot experiments on sensitiveness of different crops to sodium: A. Spinach. Plant Soil.

[19] Tomemori H., Hamamura K., Tanabe K. (2002). Interactive effects of sodium and potassium on the growth and photosynthesis of spinach and komatsuna. Plant Prod. Sci..

[20] Ors S., Suarez D.L. (2017). Spinach biomass yield and physiological response to interactive salinity and water stress. Agric. Water Manag..

[21] Xu C., Mou B. (2016). Responses of spinach to salinity and nutrient deficiency in growth, physiology, and nutritional Value. J. Am. Soc. Hortic. Sci..

[22] Suzuki N., Rivero R.M., Shulaev V., Blumwald E., Mittler R. (2014). Abiotic and biotic stress combinations. New Phytol..

[23] Zörb C., Senbayram M., Peiter E. (2014). Potassium in agriculture–Status and perspectives. J. Plant. Physiol..

[24] Nieves-Cordones M., Ródenas R., Lara A., Martínez V., Rubio F. (2019). The combination of K^+^ deficiency with other environmental stresses: What is the outcome?. Physiol. Plant..

[25] Han M., Wu W., Wu W.-H., Wang Y. (2016). Potassium transporter KUP7 is involved in K^+^ acquisition and translocation in *Arabidopsis* root under K^+^-limited conditions. Mol. Plant.

[26] Suarez D.L., Simunek J. (1997). UNSATCHEM: Unsaturated water and solute transport model with equilibrium and kinetic chemistry. Soil Sci. Soc. Am. J..

[27] Ferreira J., Sandhu D., Liu X., Halvorson J.J. (2018). Spinach (*Spinacea oleracea* L.) response to salinity: Nutritional value, physiological parameters, antioxidant capacity, and gene expression. Agriculture.

[28] Lacerda C.F., Ferreira J.F., Suarez D.L., Freitas E.D., Liu X., Ribeiro A.A. (2018). Evidence of nitrogen and potassium losses in soil columns cultivated with maize under salt stress. Rev. Bras. Eng. Agric. Ambient..

[29] Wakeel A., Abd-El-Motagally F., Steffens D., Schubert S. (2009). Sodium-induced calcium deficiency in sugar beet during substitution of potassium by sodium. J. Plant Nutr. Soil Sci..

[30] White P.J. (2013). Improving potassium acquisition and utilisation by crop plants. J. Plant Nutr. Soil Sci..

[31] Öztekin G., Uludağ T., Tüzel Y. (2018). Growing spinach (Spinacia oleracea L.) in a floating system with different concentrations of nutrient solution. Appl. Ecol. Env. Res..

[32] Sheikhi J., Ronaghi A. (2012). Growth and macro and micronutrients concentration in spinach (*Spinacia oleracea* L.) as influenced by salinity and nitrogen rates. Int. Res. J. Appl. Basic Sci..

[33] Díaz F.J., Grattan S.R., Reyes J.A., de la Roza-Delgado B., Benes S.E., Jiménez C., Dorta M., Tejedor M. (2018). Using saline soil and marginal quality water to produce alfalfa in arid climates. Agric. Water Manag..

[34] Ferreira J.F.S., Cornacchione M.V., Liu X., Suarez D.L. (2015). Nutrient composition, forage parameters, and antioxidant capacity of alfalfa (*Medicago sativa*, L.) in response to saline irrigation water. Agriculture.

[35] University of Florida (2015). Nutrient Management of Vegetable and Row Crops handbook (SP500).

[36] Nemadodzi L.E., Araya H., Nkomo M., Ngezimana W., Mudau N.F. (2017). Nitrogen, phosphorus, and potassium effects on the physiology and biomass yield of baby spinach (*Spinacia oleracea* L.). J. Plant Nutr..

[37] Cachorro P., Ortiz A., Cerdá A. (1993). Growth, water relations and solute composition of *Phaseolus vulgaris* L. under saline conditions. Plant Sci..

[38] Schroeder J.I., Fang H.H. (1991). Inward-rectifying K^+^ channels in guard cells provide a mechanism for low-affinity K+ uptake. Proc. Natl. Acad. Sci. USA.

[39] Bar-Tal A., Feigenbaum S., Sparks D.L. (1991). Potassium-salinity interactions in irrigated corn. Irrig. Sci..

[40] Ashley M.K., Grant M., Grabov A. (2006). Plant responses to potassium deficiencies: A role for potassium transport proteins. J. Exp. Bot..

[41] Anschütz U., Becker D., Shabala S. (2014). Going beyond nutrition: Regulation of potassium homoeostasis as a common denominator of plant adaptive responses to environment. J. Plant Physiol..

[42] Fageria V.D. (2001). Nutrient interactions in crop plants. J. Plant Nutr..

[43] Jakobsen S.T. (1993). Interaction between plant nutrients: III. Antagonism between potassium, magnesium and calcium. Acta Agric. Scand. Sect. B—Soil Plant Sci..

[44] Senbayram M., Gransee A., Wahle V., Thiel H. (2015). Role of magnesium fertilisers in agriculture: Plant–soil continuum. Crop Past. Sci..

[45] Feng R., Wei C., Tu S. (2013). The roles of selenium in protecting plants against abiotic stresses. Environ. Exp. Bot..

[46] Ye Y., Medina-Velo I.A., Cota-Ruiz K., Moreno-Olivas F., Gardea-Torresdey J.L. (2019). Can abiotic stresses in plants be alleviated by manganese nanoparticles or compounds?. Ecotox. Environ. Saf..

[47] Tavallali V., Rahemi M., Maftoun M., Panahi B., Karimi S., Ramezanian A., Vaezpour M. (2009). Zinc influence and salt stress on photosynthesis, water relations, and carbonic anhydrase activity in pistachio. Sci. Hort..

[48] Kronzucker H.J., Britto D.T. (2011). Sodium transport in plants: A critical review. New Phytol..

[49] Plett D.C., Møller I.S. (2010). Na+ transport in glycophytic plants: What we know and would like to know. Plant Cell Environ..

[50] Gattward J.N., Almeida A.-A.F., Souza J.O., Gomes F.P., Kronzucker H.J. (2012). Sodium–potassium synergism in *Theobroma cacao*: Stimulation of photosynthesis, water-use efficiency and mineral nutrition. Physiol. Plant..

[51] Almeida J.C.R., Laclau J.-P., Gonçalves J.L.M., Ranger J., Saint-André L. (2010). A positive growth response to NaCl applications in Eucalyptus plantations established on K-deficient soils. For. Ecol. Manag..

[52] Geilfus C.-M. (2018). Chloride: From nutrient to toxicant. Plant Cell Physiol..

[53] Franco-Navarro J.D., Brumós J., Rosales M.A., Cubero-Font P., Talón M., Colmenero-Flores J.M. (2016). Chloride regulates leaf cell size and water relations in tobacco plants. J. Exp. Bot..

[54] Szczerba M.W., Britto D.T., Kronzucker H.J. (2009). K^+^ transport in plants: Physiology and molecular biology. J. Plant Physiol..

[55] Yamada M., Kuroda C., Fujiyama H. (2016). Growth promotion by sodium in amaranthaceous plants. J. Plant Nutr..

[56] Yamada M., Kuroda C., Fujiyama H. (2016). Function of sodium and potassium in growth of sodium-loving Amaranthaceae species. Soil Sci. Plant Nutr..

