# Supplementary material for: Spinach Plants Favor the Absorption of K+ over Na+ Regardless of Salinity, and May Benefit from Na+ When K+ is Deficient in the Soil
Source: Plants (Basel). 2020 Apr 15;9(4):507. doi: 10.3390/plants9040507 (PMC7238157; doi:10.3390/plants9040507)
Supplement: Supplementary file 1 [file plants-09-00507-s001.pdf]

plants MDPI

Table S1. Concentrations of micronutrients (in mg kg^{-1} or ppm) on 'Raccoon' shoots in response to increasing concentrations of NaCl combined with two doses of potassium (K).

Nutrient Solution	'Raccoon' NaCl ($mmol_c$ L^{-1}) 5.00	30.00	60.00	90.00	120.00
**K ($mmol_c$ L^{-1})**	**Cu, mg kg^{-1}**				
**0.25**	14.49aA	8.16aA	11.33aA	9.20aA	10.77aA
**5.00**	8.12aA	5.83aA	5.25aA	4.85aA	4.74aA
	**Fe, mg kg^{-1}**				
**0.25**	511.55aA	482.70aA	632.77aA	639.59aA	521.85aA
**5.00**	456.31aA	351.07aA	361.33bA	412.18bA	299.36bA
	**Mn, mg kg^{-1}**				
**0.25**	80.69aB	98.54aA	98.96aA	94.50aA	103.10aA
**5.00**	68.36bB	72.04bAB	66.82bB	81.16bA	73.89bAB
	**Mo, mg kg^{-1}**				
**0.25**	3.34aA	2.70aB	2.18aC	2.79aB	2.93aAB
**5.00**	3.37aA	2.69aB	2.49aB	2.75aB	2.55aB
	**Se, mg kg^{-1}**				
**0.25**	3.40aA	3.30aAB	3.56aA	3.06aAB	2.81aB
**5.00**	2.03bA	2.29bA	2.18bA	2.02bA	2.45aA
	**Zn, mg kg^{-1}**				
**0.25**	26.70aA	23.50aA	26.08aA	25.23aA	32.41aA
**5.00**	24.78aA	22.60aA	20.26aA	19.97bA	21.39bA

Lowercase letters: comparisons between potassium doses, within each NaCl dose and micronutrient. Uppercase letters: comparisons between NaCl doses, within each potassium dose and micronutrient. Means following by same letter are not significantly different by Fisher's LSD test ($p < 0.05$).

Table S2. Concentrations of micronutrients (in mg kg^{-1} or ppm) on 'Gazelle' shoots in response to increasing concentrations of NaCl combined with two doses of potassium (K).

	'Gazelle'				
**Nutrient**	**NaCl ($mmol_c$ L^{-1})**				
**Solution**	**5.00**	**30.00**	**60.00**	**90.00**	**120.00**
**K ($mmol_c$ L^{-1})**	**Cu, mg kg^{-1}**				
**0.25**	6.33aAB	5.91aB	6.17aAB	6.61aAB	7.29aA
**5.00**	6.35aA	5.23aAB	4.49bB	4.59bB	4.68bB
	**Fe, mg kg^{-1}**				
**0.25**	281.96aB	338.42aAB	412.10aA	390.60aAB	428.06aA
**5.00**	369.75aB	376.16aB	274.92bB	380.79aB	509.46aA
	**Mn, mg kg^{-1}**				
**0.25**	60.83aB	75.15aA	75.92aA	72.51aA	82.44aA
**5.00**	57.82aAB	65.80aAB	57.19bB	64.16aAB	69.09bA
	**Mo, mg kg^{-1}**				
**0.25**	2.66aAB	2.24aB	2.20aB	2.48aB	2.99aA
**5.00**	2.38aA	2.02aAB	1.66bB	2.16aA	2.16bA
	**Se, mg kg^{-1}**				
**0.25**	3.05aA	3.69aA	2.67aA	2.68aA	3.16aA
**5.00**	3.12aAB	4.07aA	3.33aAB	3.82aAB	2.78aB
	**Zn, mg kg^{-1}**				
**0.25**	26.15aA	23.19aA	19.82aA	22.14aA	24.48aA
**5.00**	26.60aA	30.72aA	61.97aA	28.97aA	24.28aA

Lowercase letters: comparations between potassium doses, within each NaCl dose and micronutrient. Uppercase letters: comparations between NaCl doses, within each potassium dose and micronutrient. Means following by same letter are not significantly different by Fisher's LSD test ($p < 0.05$).

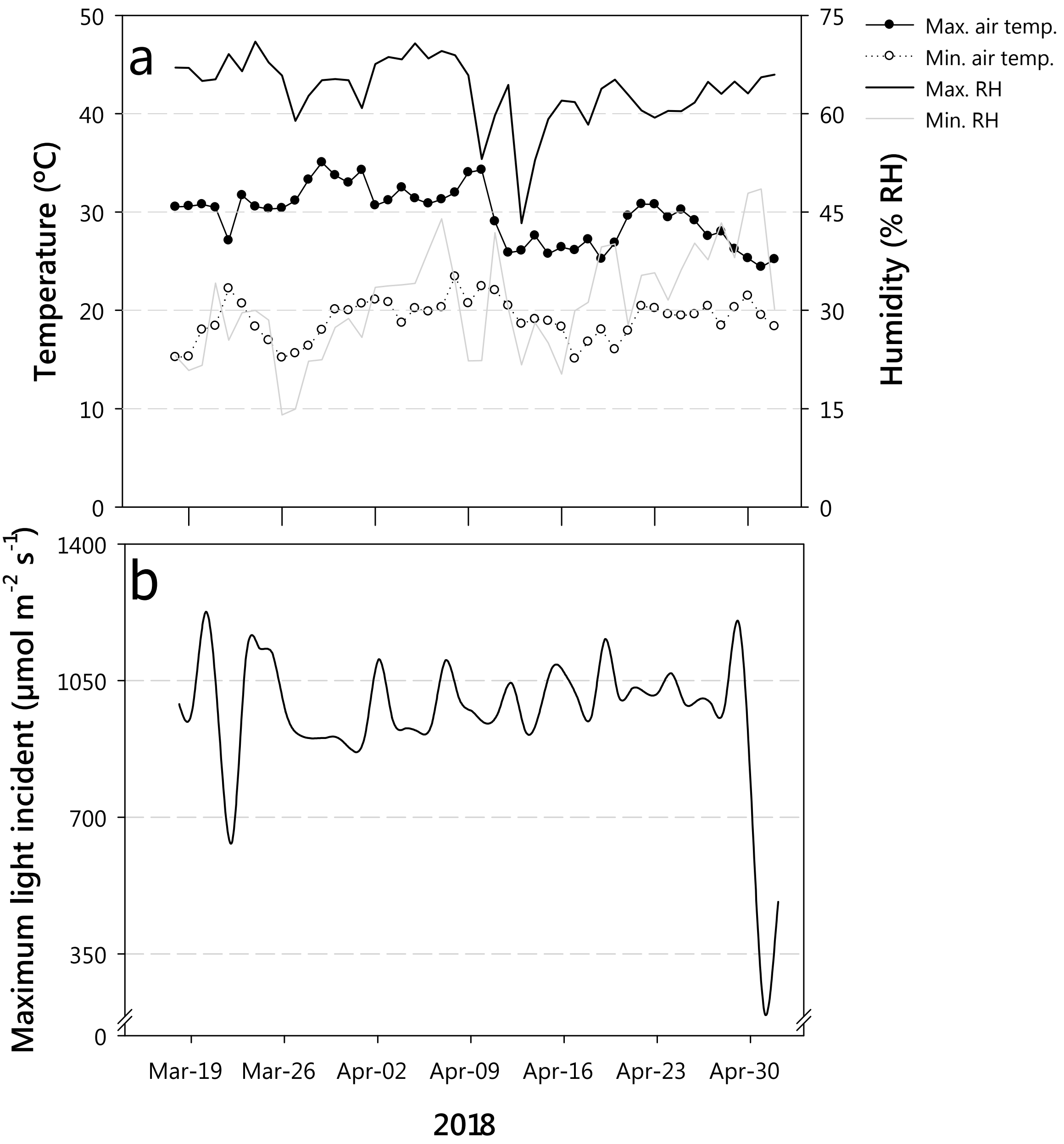

Figure S1. Temperature and humidity (a) and light intensity (b) of the greenhouse where spinach plants were cultivated from March 16 to May 15 of 2018. Intensity of natural light ranged from 650 to 1225 μmol m^{-2} s^{-1}.
